# Language Delay Is Not Predictable from Available Risk Factors

**DOI:** 10.1155/2013/947018

**Published:** 2013-03-21

**Authors:** Philip Wilson, Fiona McQuaige, Lucy Thompson, Alex McConnachie

**Affiliations:** ^1^Centre for Rural Health, University of Aberdeen, Inverness IV2 3JH, UK; ^2^Institute of Health and Wellbeing, University of Glasgow, RHSC Yorkhill, Glasgow G3 8SJ, UK; ^3^Robertson Centre for Biostatistics, Boyd Orr Building, University of Glasgow, Glasgow G12 8QQ, UK

## Abstract

*Aims*. To investigate factors associated with language delay in a cohort of 30-month-old children and determine if identification of language delay requires active contact with families. *Methods*. Data were collected at a pilot universal 30-month health contact. Health visitors used a simple two-item language screen. Data were obtained for 315 children; language delay was found in 33. The predictive capacity of 13 variables which could realistically be known before the 30-month contact was analysed. *Results*. Seven variables were significantly associated with language delay in univariate analysis, but in logistic regression only five of these variables remained significant. *Conclusion*. The presence of one or more risk factors had a sensitivity of 89% and specificity of 45%, but a positive predictive value of only 15%. The presence of one or more of these risk factors thus can not reliably be used to identify language delayed children, nor is it possible to define an “at risk” population because male gender was the only significant demographic factor and it had an unacceptably low specificity (52.5%). It is not possible to predict which children will have language delay at 30 months. Identification of this important ESSENCE disorder requires direct clinical contact with all families.

## 1. Introduction

Although there is substantial variation in the rate of language acquisition between developmentally normal individuals, most children acquire good verbal communication by the age of three years [1]. Not only is language delay among the most common developmental disorders (prevalence 1–19% depending on definition [2]) but is also an ESSENCE disorder [3] commonly associated with negative long-term outcomes [4–6]. These include social and behavioural problems, lack of school readiness [7], school exclusion [8], future academic problems [9], neuropsychiatric disorders [10], and poor employment [11]. 

A number of studies (e.g., [4, 12]) have supported the argument that early interventions can affect long-term outcomes, but there are many methodological weaknesses in trial design [13], and findings of trials based on community screening are inconsistent [13, 14]. There has thus been no international consensus to date on the wisdom of screening for language delay. There is no screening programme currently in the UK, largely because of the lack of historical evidence of effectiveness [15, 16]. The evidence base has, however, developed substantially in the past decade. Miniscalco et al. [17] evaluated a simple Swedish language screening instrument and found that it accurately identified language delay in 2.5-year-old children. Further, a cluster randomised trial of language screening for toddlers in The Netherlands concluded that screening can reduce the number of children who require special education and leads to improved language performance at age eight [18]: the authors recommended nationwide implementation of the screening instrument. Contrasting conclusions have emerged from recent work in Australia [14]. There remain significant methodological challenges to the development and adoption of a universally accepted screening instrument [19].

Child health screening activity for the whole population has been substantially curtailed in the UK [16] and in Scotland at the time of writing there is currently no universal health surveillance contact for children beyond 16 weeks of age [20]. Before 16 weeks, families are usually visited at home on a number of occasions by their community child health nurse (health visitor) who offers support and assesses developmental or social vulnerabilities. After the neonatal medical examination, there is only one scheduled developmental assessment, at six weeks of age. Parents are nevertheless free to contact their general medical practitioner or their health visitor if they have concerns about a child's development. The arguments for the dismantling of a universal child health surveillance system were based upon both lack of evidence of effectiveness [16] and social inequity. This latter issue is often described as the “Inverse Care Law” [21]: the selective uptake of preventative health services by those who least need them. The withdrawal of universal developmental screening in Scotland was predicated upon assumptions that appropriately informed parents would attend services with concerns about their children's development, in tandem with the view that high risk children could be identified by methods other than universal routine health checks: for example, because of social deprivation, involvement of hospital services, or through the early postnatal assessments carried out by health visitors [20]. 

The Scottish model for supporting early child development is thus founded on two principles: parental awareness and targeted surveillance based on known risk factors. We have recently reported on pilot work carried out by health visitors with 30-month-old children in West Glasgow [22] and demonstrated that a substantial proportion of developmental problems had not hitherto been suspected, raising concerns about reliance on parental awareness as a trigger to service contact.

In the present paper, we test the second assumption that known risk factors can be used by child health nurses to predict the key ESSENCE disorder, language delay.

## 2. Methods

Health visitors in West Glasgow Community Health and Care Partnership were asked to visit all families in their caseload when their child reached the age of 30 months. Details of the population base and the organisation of this visit are given in Thompson et al. [22]. At the contact, health visitors completed three questionnaires with the principal carer of the child (usually the mother):

- the Richman Behaviour Checklist [23], a list of 21 problematic childhood behaviours scored as 0, 1, or 2;
- the Parenting Daily Hassles Scale (PDHS) [24], which lists 20 perceived parental stresses, each scored both in terms of frequency and severity;
- a language screen consisting of two questions [17]:
  - can your child put two or more words together?
  - can your child say at least 50 words?

The language screen is a modification of Miniscalco's screening instrument: a vocabulary of fewer than 50 words at 30 months was found to be a reasonable indication of language delay, with a sensitivity of 0.69 and specificity of 0.93 [17]. 

The health visitors were asked to record other information, including but not limited to the following.

- Any existing medical problems with the child or other family members. For the sake of brevity, this question did not go into further detail so items were recorded entirely at the discretion of the health visitor.
- Details of service provision to date.
- HPI (health plan indicator) status [25]; each child is assigned by the health visitor to Core, Additional, or Intensive status which indicates the level of continued contact needed. For most Scottish children, the HPI status would have been allocated in the first year of life and not reconsidered thereafter [26]. Children assigned to the Core category would not normally be seen by the health visitor on a planned routine basis.
- Details of who lives with the child.

No more detailed examination of the child was performed on a routine basis.

The data collection sheet is provided in Appendix A. Information collected from these contacts along with Scottish Index of Multiple Deprivation (SIMD) rankings for the datazones of residence of the family [27] were collated for analysis. SIMD is an area-based measure of deprivation referenced to the whole Scottish population: Glasgow has a relatively high level of deprivation and about half of our sample is in the most deprived Scottish SIMD quintile. This study used SIMD data from 2009, the year of data collection. Health visitors were able to insert free text on the data collection sheet including, in some cases and at their discretion, whether the family used more than one language at home. The potential predictor variables that we used in our analyses thus correspond to those that a health visitor might reasonably be expected to be able to access for a child who had not been seen since infancy.

### 2.1. Statistical Analysis

Disagreement with the “can your child say at least fifty words” statement was used to represent presence of language delay. All the children reported to be unable to make two-word utterances were also reported as being unable to say 50 words.

Thirteen potential predictor variables for language delay which were potentially available to the health visitor could feasibly have been known before the 30-month contact. They include demographic, service use and personal and family medical history and are listed in Appendix B. Univariate associations were tested using Fisher's exact tests. Those variables that showed some evidence (*P* < 0.1) of association with language delay were entered into a multiple logistic regression model, and a backward stepwise procedure was used to derive a model including only those factors showing an independent association with language delay at a 5% significance level. The diagnostic performance of the number of predictive factors was assessed in terms of sensitivity, specificity, and positive predictive value.

Ethics committee review was not required for this piece of work as it formed part of an NHS service evaluation.

## 3. Results

Three hundred and thirty families (40% of 819 eligible) received a visit and data for the language screen were available for 315 children (95% of the 330 visited). Language delay, defined as reported inability to say 50 words, was evident in 33 children (10.5% of 315).  Table 1 shows the prevalence of language delay in relation to the potential predictor variables. There was no evidence (*P* > 0.1) that language delay using our definition was associated with deprivation (SIMD quintile), known problems with alcohol or drug abuse in the family, involvement with social work services, the father not being at home, or parental mental illness.

Only two children had an involvement with the Community Paediatrics Team, and both showed signs of language delay (*P* = 0.011). This variable would not, however, have any value in a logistic regression model due to the small number of children with the factor. Consequently, we combined this indicator with “Involvement with Other Services,” which was also positively associated with language delay (*P* = 0.018), to create a variable “Involvement with non-Social Work Services” to be used in the logistic regression analysis. This factor identified 24 children, of whom 8 (33%) were positive on the language delay screen, compared to 25/291 (8.6%) without this factor (*P* = 0.001).

Table 2 reports the results of logistic regression modelling. Attendance at nursery and HPI status at the start of the visit did not show evidence of independent associations with language delay. Language delay was independently associated with male gender, involvement with services other than social work, behavioural and developmental problems of the child or the family, and with bilingual families.

**Figure 1:**
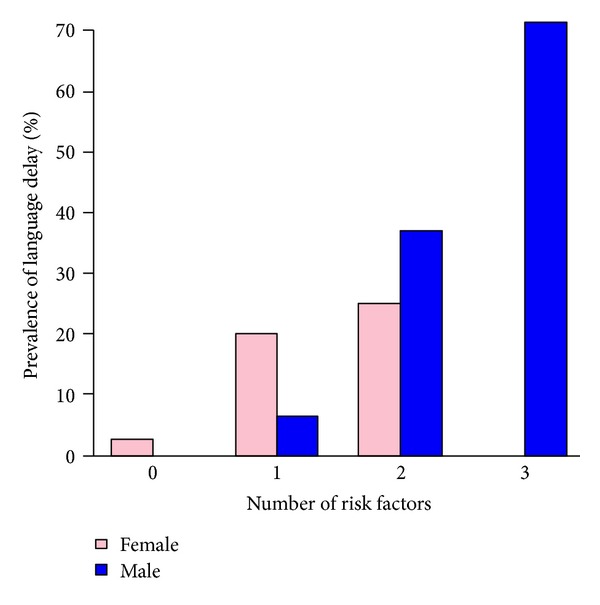
Prevalence of language delay at 30 months in relation to number of risk factors, by gender.

Table 3 and Figure 1 show the prevalence of language delay in relation to the number of risk factors identified by logistic regression, overall and separately for boys and girls. There was a strong association between the number of risk factors and language delay at 30 months. Whilst the presence of one or more risk factors had a sensitivity of 89%, this threshold included all male children, and the specificity was low, at 45%: more importantly, the positive predictive value was only 15%. The presence of two or more risk factors had a specificity of 93%, but a sensitivity and positive predictive value of only 48% and 43%.

## 4. Discussion

We first aimed to establish which preexisting factors are significantly associated with language delay at 30 months. Five predictor variables were identified; male gender, involvement with services other than social work, behavioural and developmental problems of the child or the family, and living in a bilingual household. Given the lack of universal child health screening contacts in Scotland, we also sought to establish whether preexisting data could be used to identify children at risk of language delay with an acceptable degree of accuracy.

The association of language delay with “involvement with services other than social work” variable is unsurprising. The number of such children was relatively small (24; 8%) and the variable covers a wide range of services which were not individually specified. It is likely that at least some types of service use (e.g., community paediatric services) are already used by nurses in their approaches to identification of developmental vulnerability.

Our finding of an association between being in a bilingual household and language delay must be considered tentative. Previous studies have noted that bilingual children can be at a greater risk of either being misdiagnosed with language difficulties, or of being overlooked because language problems in this group are difficult to be diagnosed accurately [28]. Problems of reporting bias may also have influenced the data on bilingualism: health visitors were not specifically asked to report on bilingualism and may have done so more readily if the child had language delay. These findings need confirmation in a more robust design.

The remaining predictive factors are male gender and preexisting behavioural and developmental problems in either the child or the family. The utility of both of these categories in the identification of children at risk for developmental delay is doubtful. Behavioural and developmental problems do not at present meet UK national screening criteria and consequently screening is not offered [29], although there may be an increasingly strong case for screening for persistent conduct disorder [30]. As there is no reliable method of identifying developmental and behavioural problems without some sort of assessment of the child or family, it is not feasible to use knowledge of preexisting behavioural and developmental problems in a targeting strategy to identify the children at high risk of language delay. 

While using gender as a predictive tool would be easy, and there is a significant association between male gender and language delay, the utility of this predictor is clearly limited: many girls have language delay and selective screening of boys would clearly be discriminatory.

Each of the predictive factors identified in this study thus has flaws which make them unsuitable for use as screening tools. Furthermore, 11.1% of the children with language delay had no risk factors (including male gender) and a significant proportion of children with language delay would be missed, even with “selective” targeting of most of the population.

### 4.1. Strengths and Limitations of the Study

Although our sample consisted of under half (40%) of the eligible population, our analyses of the missing data suggest that most was due to differential engagement of the staff in the pilot area [22, 31] rather than nonparticipation by families. As well as good representativeness in terms of socioeconomic status, our sample's preexisting risk assessment (HPI) categories did not differ significantly from the population, with most families in the core category [22]. While our sample was representative of the population of the area in terms of socioeconomic status and HPI, it may have differed from the whole population on unmeasured variables. The fact that this study was carried out in the context of a service evaluation, rather than a research project, may have improved the generalizability of our findings.

Relatively few potentially predictive variables were available for analysis: for example, details of family history and household language were not recorded systematically in most cases. Because this was a service evaluation, not a research project, it is likely that families were not asked the questions about language delay in a consistent way. The language screen itself was very basic and it is possible that questions about receptive language ability may have been more sensitive in identifying all children with verbal communication problems. Nevertheless, we consider it likely that the majority of the children (10.5% of the whole sample) would have significant verbal communication problems. Our sample was nevertheless relatively small which may have impacted on the outcome of multivariate analyses. 

### 4.2. Comparison with the Existing Literature

When comparing this study's results with those in previous literature, the association between behavioural and developmental problems and language delay is not unexpected: it has been consistently demonstrated over the years. A cross-sectional study [32] in a London borough in the 1970s found that 58% of the language delayed children had behaviour problems compared to only 14% of the nonlanguage delayed children. A decade later, Baker and Cantwell [33] also reported that children with language difficulties had a high rate of emotional and behavioural problems. More recently Van Daal et al. [34] found that 40% of children with language impairment displayed serious significant behaviour problems. More detail of the overlap between reported behavioural problems and language delay in the sample reported here is given in the study of Thompson et al. [22]. Our finding that language delay was independently associated with male gender is also supported by many previous studies (e.g., [35, 36]).

Several authors have reported a significant association between socioeconomic deprivation and delayed language development. This association has been attributed to several interlinked factors: for example, maternal educational levels (and consequently vocabulary) are generally greater in higher socioeconomic groups, and rates of maternal depression, drug, and alcohol misuse are greater in more deprived socioeconomic groups [37, 38].

The present study is not unique, however, in finding no apparent association between language delay and socioeconomic status or factors associated with lower socioeconomic status, that is, family mental health problems and family drug or alcohol misuse. Other studies have had similar results: Berglund et al. [35] and Choudhury and Benasich [36] both found that socioeconomic status was not significantly related to language ability. This indicates that it is entirely possible that socioeconomic status is unrelated to abnormal language development in West Glasgow, although it is likely that the range of normal language development would vary with maternal educational attainment [39]. In line with O'Callaghan et al. [37], we found that marital status of the child's parents was unrelated to language delay.

Berglund et al. [35] reported that children who attended day-care centres had higher language abilities than those who did not. In our univariate analysis, attending nursery was significantly associated with a lower rate of language delay, but this association became nonsignificant after adjustment for confounders such as socioeconomic status.

## 5. Conclusions and Recommendations

It is not feasible to use the presence of preexisting available risk factors to identify language delay at 30 months with any reasonable degree of accuracy. It is also not possible to define an “at risk” population group because, apart from the poorly predictive association with male gender, there were no demographic factors significantly associated with language delay. Previous studies have come to similar conclusions; Baker and Cantwell [33], Zubrick et al. [40], Reilly et al. [39], and Schjølberg et al. [41] found no demographic variables which could realistically be used to identify high risk children. Our findings, which add variables related to services use and risk category allocated in infancy to demographic predictors, provide strong support for the view that universal language screening programs are the only effective way of identifying children with language delay. 

It appears that the use of specific questions about language delay, rather than simply asking parents if they are concerned about their child's language development, is necessary. Miniscalco et al. [17] and others reported that parental concern is not a reliable guide to language skills in toddlers and Westerlund and Sundelin [42] found that only 64% of the 3-year-old children in their study with language delay would have been identified by parental concern alone.

We think that there is a compelling case for community child health services to approach all families with children who aged two years. A finding of language delay should trigger further assessment of motor function, social communication, attention, hyperactivity, and overall cognitive performance—the ESSENCE disorders [3]. Since the work reported in this paper was conducted, the Scottish Government has reintroduced a universal child health screening contact, focussed on language, behaviour, and social development, at 27 months [43].

## Supplementary Material

Data collection cover sheet. This form was completed by health visitors before and after making the visit to families with 30-month-old children.

## Figures and Tables

**Table 1:** Univariate analysis. Prevalence of language delay at 30 months in relation to potential risk factors, with Fisher's exact test *P* values.

	*N*	*N* (%) with language delay	*P* value
SIMD quintile (26 missing)			
Q 1	121	17 (14.0%)	*P* = 0.342
Q 2	42	4 (9.5%)	
Q 3	47	4 (8.5%)	
Q 4	26	3 (11.5%)	
Q 5	53	2 (3.8%)	
Attends nursery (2 missing)			
No	178	25 (14.0%)	*P* = 0.025
Yes	135	8 (5.9%)	
Is there any known problem with drug or alcohol use in the family? (4 missing)			
No	294	28 (9.5%)	*P* = 0.233
Yes	17	3 (17.6%)	
HPI status at start of visit			
Core	200	14 (7.0%)	*P* = 0.012
Additional	84	12 (14.3%)	
Intensive	31	7 (22.6%)	
Involvement with community paediatrics team			
No	313	31 (9.9%)	*P* = 0.011
Yes	2	2 (100.0%)	
Social work involvement			
No	296	30 (10.1%)	*P* = 0.434
Yes	19	3 (15.8%)	
Involvement with other services			
No	293	27 (9.2%)	*P* = 0.018
Yes	22	6 (27.3%)	
Gender (26 missing)			
Female	146	10 (6.8%)	*P* = 0.054
Male	143	20 (14.0%)	
Father not at home (2 missing)		
No	258	28 (10.9%)	*P* = 0.813
Yes	55	5 (9.1%)	
Child's behavioural and developmental problems (7 missing)			
No	296	25 (8.4%)	*P* < 0.001
Yes	12	7 (58.3%)	
Parental mental illness (10 missing)			
No	288	28 (9.7%)	*P* = 0.396
Yes	17	3 (17.6%)	
Familial behavioural and developmental problems (9 missing)			
No	297	28 (9.4%)	*P* = 0.052
Yes	9	3 (33.3%)	
Bilingualism (bilingual family)			
No	288	26 (9.0%)	*P* = 0.014
Yes	27	7 (25.9%)	

**Table 2:** Multivariate analysis. Effects of candidate predictor variables, reported as odds ratio for language delay with 95% confidence interval and *P* value.

Predictor	Model 1	Model 2
	Estimate (95% CI), *P* value	Estimate (95% CI), *P* value
Attends Nursery		
Yes versus no	0.53 (0.20, 1.44), *P* = 0.212	
HPI status at start of visit		
Additional versus core	0.82 (0.25, 2.70), *P* = 0.740	
Intensive versus core	1.02 (0.21, 4.93), *P* = 0.979	
Involvement with non-SW services		
Yes versus no	4.58 (1.16, 18.10), *P* = 0.030	4.31 (1.25, 14.86), *P* = 0.021
Gender		
Female versus male	2.90 (1.06, 7.89), *P* = 0.038	2.66 (1.00, 7.11), *P* = 0.050
Child's behavioural and developmental problems		
Yes versus no	8.26 (1.73, 39.43), *P* = 0.008	8.02 (1.89, 33.97), *P* = 0.005
Family behavioural and developmental problems		
Yes versus no	6.06 (0.87, 42.40), *P* = 0.069	6.85 (1.07, 43.82), *P* = 0.042
Bilingual Family		
Yes versus no	5.62 (1.76, 18.01), *P* = 0.004	5.89 (1.87, 18.57), *P* = 0.003

Model 1: all predictors with *P* < 0.1 at univariate analysis. Model 2: best fitting model found by backwards selection, starting with model 1, with stepwise exclusion of terms with *P* > 0.05.

**Table 3:** Prevalence of language delay at 30 months in relation to number of risk factors, overall and by gender, with Fisher's exact test *P* values. *N* = 273 (27 with language delay) after excluding 32 children with missing data for one or more risk factors.

	Number of risk factors				*P* value
	0	1	2	3	
Overall					
No language delay	111	118	15	2	*P* < 0.001
Language delay	3	11	8	5	

Prevalence	2.6%	8.5%	34.8%	71.4%	

Males					
No language delay	0	102	12	2	*P* < 0.001
language delay	0	7	7	5	

Prevalence	—	6.4%	36.8%	71.4%	

Females					
No language delay	111	16	3	0	*P* = 0.005
Language delay	3	4	1	0	

Prevalence	2.6%	20.0%	25.0%	—	

## Appendices

### A. The Visit Cover Sheet

See supplementary material available online at http://dx.doi.org/10.1155/2013/947018.

### B. Variables Tested for Association with Language Delay

*Continuous Variable*

*SIMD Rank*. Scottish index of multiple deprivation ranking for each child's household.

*Categorical Variables*. The following categorical variables were all derived from yes/no answers to the following questions.

- Is the child attending nursery?
- Is there any known problem with drug or alcohol use in the family?
- Are there any 1st degree relatives not living within the household?
- Are there any significant diagnoses (in the child) with long-term implications for the child's development?
- Is there any relevant family medical history likely to have an impact on the child's development?
- HPI status at start of visit.
- Involvement with community paediatric team.
- Social work involvement.
- Involvement with other services.
- Gender.

*New Categorical Variables. *Three of the categorical variables had additional details provided in the dataset that were used to create new more specific variables.

- Father not at home.

From the “first degree relatives not living within the household” variable a “father not at home” variable was created.

- Child's behavioural and developmental problems.
- Child medical conditions with child's behavioural developmental problems not included.

From the “significant diagnoses for the child” variable a “child's behavioural and developmental problems” variable was created.

- Parental mental illness.
- Familial behavioural and developmental problems.
- Family medical history with familial behavioural and developmental problems not included.

From the “relevant family history” variable “parental mental illness” and “familial behavioural and developmental problems” variables were created.

- Bilingual family.

The free text in the dataset comprised information that the health visitors felt was noteworthy. From this information it was clear that several children came from bilingual families, so this information was used to create a new bilingual variable.
