# Supplementary material for: Language Delay Is Not Predictable from Available Risk Factors
Source: ScientificWorldJournal. 2013 Mar 21;2013:947018. doi: 10.1155/2013/947018 (PMC3618945; doi:10.1155/2013/947018)
Supplement: Supplementary file 1 — Data collection cover sheet. This form was completed by health visitors before and after making the visit to families with 30-month-old children. [file 947018.f1.pdf]

Child Surveillance Contact @ 30 months

Child's name [ ] CHI number [| | | | | | | | |]

Postcode [| | | | | |]

Date of contact [d|d|m|m|y|y] Length of contact [hrs|mins]

HV number [ ] CHCP [ ]

Have you received Solihull training? Yes [ ] No [ ]

If yes, date trained [d|d|m|m|y|y]

HPI before contact: Core [ ] Additional [ ] Intensive [ ]

HPI after contact: *(please record even if there is no change)* Core [ ] Additional [ ] Intensive [ ]

Was this contact: Face-to-face [ ] By phone [ ] By post [ ] Combination [ ]

For face-to-face, were questionnaires sent in advance? Yes [ ] No [ ]

Where sent, did parent complete questionnaires in advance? Yes [ ] No [ ]

Did you experience any problems in setting up this contact or getting questionnaires back? Yes [ ] No [ ]

If yes, please give details

Are there any significant diagnoses with long term implications for child's development?

No [ ] Yes [ ] *Please describe:*

Is the child/family involved with any of the following services?

Social work services [ ] PACT team [ ]

Community paediatrics [ ] Other [ ] *Please state:*

Is the child attending nursery?

No [ ] Yes [ ] *How many hours per week (on average)?*

Who lives in the home with the child? *Please list all:*

PTO

Are there any first degree relatives not living within the household? (e.g., father, mother or siblings)?

No ☐ Yes ☐ — Please list all:

Is there any relevant family medical history likely to have an impact on the child's development?

No ☐ Yes ☐ — Please describe:

Is there any known problem with drug or alcohol use in the family?

No ☐ Yes ☐ — Please describe:

Is there anything else you would like to comment on?
(*Is there anything in this person's social environment or past experiences that you feel may compromise their parenting role? E.g., experience of violence in the household, substance misuse, financial or housing worries, mental health issues, experience of discrimination, language or communication support needs.*)

Action Plan

- ☐ No action required
- ☐ Revisit (state when):
- ☐ Referral (state where):

Practitioner signature: ______________________________
